# Tinnitus risk after COVID-19 XBB.1.5 vaccination: A self-controlled case series study

**DOI:** 10.1016/j.vaccine.2025.127548

**Published:** 2025-07-26

**Authors:** Stanley Xu, Lina S. Sy, Vennis Hong, Lei Qian, Kimberly J. Holmquist, Katia J. Bruxvoort, Bing Han, Bruno Lewin

**Affiliations:** a Department of Research & Evaluation, Kaiser Permanente Southern California, Pasadena, CA, USA; b Department of Health Systems Science, Kaiser Permanente Bernard J. Tyson School of Medicine, Pasadena, CA, USA; c School of Public Health, University of Alabama at Birmingham, Birmingham, AL, USA; d Department of Clinical Science, Kaiser Permanente Bernard J. Tyson School of Medicine, Pasadena, CA, USA

**Keywords:** Tinnitus, COVID-19 vaccination, COVID-19 XBB.1.5, Influenza vaccination, Coadministration, Self-controlled case series

## Abstract

**Background:**

While prior studies found no clear evidence of an association between COVID-19 vaccination and tinnitus, concerns about a potential link remain. This study assessed the risk of tinnitus following COVID-19 XBB.1.5 vaccination, administered alone or coadministered with influenza vaccine, using the event-dependent self-controlled case series (SCCS) design.

**Methods:**

We conducted an SCCS study among individuals aged ≥12 years enrolled at Kaiser Permanente Southern California, with tinnitus events occurring between September 1, 2023, and March 31, 2024. The exposures included Pfizer-BioNTech and Moderna COVID-19 XBB.1.5 vaccines, with or without influenza vaccine coadministration. The primary outcome was first-ever tinnitus (no documented history before September 1, 2023), and the secondary outcome was first-in-1-year tinnitus, identified using ICD-10 code H93.1* in inpatient, emergency department, and outpatient settings. Risk intervals were pre-specified as 1–14 days and 1–28 days after vaccination, with person-time outside the risk interval serving as the control interval. Relative incidences (RI) and 95% confidence intervals (CI) were estimated, adjusting for seasonality by including tinnitus events among non-recipients of COVID-19 XBB.1.5 vaccines and by including calendar month in the SCCS models.

**Results:**

With 13,940 first-ever tinnitus events among recipients of COVID-19 XBB.1.5 vaccines, no increased risk was observed within 1–14 or 1–28 days following vaccination in overall analyses. The RI was 0.78 (95% CI: 0.67–0.90) for the 14-day risk interval and 0.87 (95% CI: 0.78–0.96) for the 28-day interval. Subgroup analyses by age and influenza vaccine coadministration status also showed no significant increase in RI. Similarly, no significantly elevated RI was found for first-in-1-year tinnitus in the overall analyses, age-specific analyses, or analyses by coadministration with influenza vaccine.

**Conclusion:**

Our findings suggest no increased tinnitus risk following COVID-19 XBB.1.5 vaccination, either administered alone or coadministered with influenza vaccine. These results provide reassuring evidence of the safety of COVID-19 vaccines with respect to tinnitus risk.

## 1. Background

COVID-19 vaccination has undergone extensive safety evaluation in the United States (U.S.) and globally, with numerous studies confirming its favorable safety profile. Research has consistently shown that serious adverse events following vaccination, including myocarditis, pericarditis, Guillain-Barré syndrome (GBS), thrombotic events, and mortality, are exceedingly rare. For example, studies reported that risk of myocarditis and pericarditis increased after mRNA COVID-19 vaccines, primarily observed in young males [1–4]; however, these myocarditis and pericarditis events occurred at rates lower than those associated with SARS-CoV-2 infection itself [5,6]. Additionally, an increased risk of thrombosis with thrombocytopenia syndrome and GBS following adenoviral vector COVID-19 vaccines was observed, contributing to the discontinuation of these vaccines worldwide [7–12]. Further, several studies evaluated mortality following COVID-19 vaccination and found no increased risk of all-cause death, non-COVID-19 mortality, and cardiac-related mortality [13–17]. These evaluations have guided vaccination policies worldwide, ensuring that the benefits of COVID-19 vaccination in preventing severe illness, hospitalization, and death far outweigh the risks of rare adverse events.

Case reports of less severe concerns post COVID-19 vaccination have been reported for other medical conditions including tinnitus [18–20]. Tinnitus is a medical condition characterized by the perception of sound in the absence of external auditory stimuli. It affects approximately 10 % of the U.S. population, presenting as ringing, chirping, humming, or other persistent noises in the ears [21]. Tinnitus can be classified into two types: subjective tinnitus, perceived only by the affected individual, and objective tinnitus, detectable by a healthcare provider using specialized instruments. Subjective tinnitus is the more common form. During December 14, 2020–May 4, 2023, VAERS received 17,859 reports of tinnitus after COVID-19 vaccination [22]. Although these occurrences represent a small fraction of vaccinated individuals, the potential association has prompted investigation.

Given concerns about a possible increased risk of tinnitus after COVID-19 vaccination, several observational studies have been conducted to examine this issue. Findings have been mixed. For example, a retrospective chart review at a tertiary otolaryngology practice found no definitive link between COVID-19 vaccination and tinnitus [23]. Similarly, a study that analyzed VAERS and Vaccine Safety Datalink (VSD) data concluded that tinnitus incidence rates following COVID-19 vaccination were similar to those after influenza vaccination [22]. However, other studies have suggested a potential association. One study found that pre-existing metabolic conditions, such as hypertension and obesity, were correlated with the severity of tinnitus symptoms post-vaccination [24]. The same study also noted that tinnitus onset was often rapid, with nearly half of the cases occurring within two days of vaccination, and that tinnitus was associated with other neurological and psychiatric symptoms.

A systematic review of the literature concluded that while the incidence of tinnitus following COVID-19 vaccination is rare, there is biological plausibility for a vaccine-associated mechanism, including molecular mimicry and immune-mediated responses [25]. The review also emphasized the need for further research, particularly in individuals with pre-existing autoimmune or otologic conditions. Additionally, a self-controlled case series (SCCS) study conducted in Australia found a statistically significant increased risk of tinnitus in the 42 days following both the adenoviral vector (Vaxzevria^®^) and mRNA COVID-19 vaccines, with relative incidences of 2.25 and 1.53, respectively [26]. This study, which identified tinnitus in a general practice setting, was among the first to demonstrate an association between tinnitus and COVID-19 vaccination using population-level healthcare data. However, it did not evaluate the XBB.1.5 monovalent vaccine specifically and did not adjust for seasonality, and potential confounding by concurrent SARS-CoV-2 infection could not be ruled out. These findings underscore the variability in the existing literature and highlight the importance of continued evaluation.

The objective of this study was to assess the risk of tinnitus following administration of updated COVID-19 vaccine targeting the XBB.1.5 variant (COVID-19 XBB.1.5 vaccine) using a SCCS design [27–29]. This study design accounts for time-invariant confounders by comparing the incidence of tinnitus in predefined risk periods after vaccination to control periods within the same individuals.

## 2. Methods

### 2.1. Study population and study period

This SCCS study utilized the electronic health records of a cohort of individuals aged ≥12 years enrolled at Kaiser Permanente Southern California (KPSC), a large health care system serving over 4.8 million patients across 15 medical centers and approximately 250 medical offices throughout Southern California. The race or ethnicity distribution, demographics, and socioeconomic profiles of KPSC health plan enrollees are comparable to those of Southern California residents [30]. The SCCS analytic datasets included individuals who experienced tinnitus events between September 1, 2023, and March 31, 2024. Individuals were required to have at least one year of continuous enrollment (allowing for a 31-day gap) prior to September 1, 2023. The KPSC Institutional Review Board approved the study (IRB #13270) and waived the requirement for informed consent due to the study posing minimal risk to participants.

### 2.2. Exposure and observation period

The exposures included the administration of Pfizer-BioNTech or Moderna COVID-19 XBB.1.5 vaccines and coadministration with influenza vaccine during the period from September 1, 2023, to March 31, 2024. The observation period for vaccine recipients started on September 1, 2023, and ended on March 31, 2024, or upon death, receipt of a second dose of COVID-19 XBB.1.5 vaccine, or disenrollment, whichever occurred first.

### 2.3. Outcome definition

Tinnitus events were identified using International Classification of Diseases, Tenth Revision (ICD-10) code H93.1* [22] in inpatient, emergency department (ED), or outpatient settings. The primary outcome was first-ever tinnitus, defined as tinnitus occurring in an individual with no documented history of tinnitus since October 1, 2015 and before September 1, 2023. The secondary outcome was first-in-1-year tinnitus, defined as tinnitus occurring in an individual with no documented history of tinnitus in a one-year period prior to the first tinnitus diagnosis date during the observation period. We considered these tinnitus events that were identified with ICD-10 codes to be electronically identified tinnitus events. Tinnitus event dates were defined as the dates on which a tinnitus diagnosis was recorded during a medical encounter, rather than the actual date of symptom onset because symptom onset dates were not available in structured electronic health record (EHR) data and would have required extensive manual chart review.

### 2.4. Statistical analyses

Demographic characteristics of individuals who received the COVID-19 XBB.1.5 vaccine and experienced tinnitus during the study period were described separately for first-ever and first-in-1-year tinnitus events. The event-dependent SCCS approach tailored for event-dependent exposures was employed to estimate relative incidence (RI) and 95% confidence intervals (CI), comparing the risk intervals to the corresponding control intervals [31]. The risk intervals were pre-specified as 1–14 days and 1–28 days after vaccination in our primary SCCS analyses, with person-time outside of these risk intervals serving as the control interval. In this context, “risk interval” refers to the time period during which the event is hypothesized to be associated with the exposure, while the “control interval” refers to the period not considered at risk, serving as a baseline for comparison. We also assessed tinnitus risk for two longer risk intervals: 1–42 days and 1–90 days after vaccination in sensitivity analyses. To adjust for the seasonality of tinnitus, we also included unvaccinated cases among individuals who did not receive COVID-19 XBB.1.5 vaccines and experienced tinnitus during the observation period. Depending on the outcomes to be analyzed, we used either first-ever tinnitus or first-in-1-year tinnitus among the unvaccinated. Calendar month was included in the SCCS analyses to account for potential temporal trends. Subgroup analyses were performed by age (<40 years, 40–59 years, and ≥ 60 years) and by coadministration with influenza vaccine on the same day (yes/no). Age was not adjusted as a time-varying covariate given the relatively short observation period of seven months.

Chart reviews of tinnitus cases were planned for two purposes: 1) to assess the temporal relationship between the tinnitus event and vaccination for tinnitus cases occurring on the vaccination date (Day 0); and 2) to enable re-analyses of confirmed cases if a significantly elevated RI was observed in analyses of electronically identified tinnitus events. A chart review of a random sample of 20 tinnitus cases on Day 0 showed that all events occurred before the COVID-19 XBB.1.5 vaccination; therefore, tinnitus cases occurring on the vaccination date were excluded from calculating relative incidences and risk intervals began on Day 1. In practice, when using the R package for the event-dependent SCCS, we treated Day 0 as a separate risk interval but did not report its RI, following the recommendation by Schultze et al. [32]. Since no safety signal (i.e., the lower bound of the 95% CI for RI exceeding 1.0) was detected in analyses of electronically identified tinnitus events, no additional chart reviews were conducted.

SARS-CoV-2 infection may influence the observed association between vaccination and tinnitus. To address this, we conducted a sensitivity analysis excluding tinnitus events that occurred within 30 days of a documented SARS-CoV-2 infection (based on a positive test or a COVID-19 diagnosis) in both the first-ever and first-in-1-year tinnitus analyses [14,33].

Analyses were conducted using SAS version 9.4 (SAS Institute), and the event-dependent SCCS models were fitted using the R package (R Core Team) SCCS [34]. A sample of the R code used for our SCCS analyses is provided in the Appendices.

## 3. Results

### 3.1. Demographic characteristics of recipients of COVID-19 XBB.1.5 vaccines who experienced tinnitus

Among the 13,940 recipients of COVID-19 XBB.1.5 vaccines who experienced first-ever tinnitus, 49.7 % were aged 60 years or older, and there was a slightly higher proportion of females (53.0 %) compared to males (47.0 %) (Table 1). Most cases occurred among Non-Hispanic White (35.7 %) and Hispanic (42.2 %) individuals, while Non-Hispanic Asian/Pacific Islanders and Non-Hispanic Black individuals represented 10.8 % and 6.7 % of tinnitus cases, respectively. Most tinnitus cases (97.0 %) were identified in the outpatient setting, with only 0.5 % and 2.5 % occurring in inpatient and ED settings, respectively.

Similarly, 17,875 recipients of COVID-19 XBB.1.5 vaccines experiencing first-in-1-year tinnitus had comparable demographic characteristics (Table 2). The largest age group was those aged 60 years or older (53.2 %), with females accounting for 53.0 % of tinnitus cases. The racial/ethnic distribution was similar, with Non-Hispanic White (35.6 %) and Hispanic individuals (42.4%) making up the majority of tinnitus cases. Most tinnitus cases (97.1 %) were detected in outpatient settings, with inpatient and ED settings comprising 0.5 % and 2.4 % of tinnitus cases, respectively.

### 3.2. First-ever tinnitus

In the overall analyses, the RI of first-ever tinnitus after COVID-19 XBB.1.5 vaccination was 0.78 (95% CI: 0.67–0.90) in the 1–14 day risk interval and was 0.87 (95% CI: 0.78–0.96) in the 1–28 day risk interval, indicating no association of COVID-19 XBB.1.5 vaccination with increased risk of first-ever tinnitus (Table 3). For the 1–14 day risk interval, the RI among those with influenza vaccine coadministration was 0.93 (95% CI: 0.70–1.24) in the overall analyses, while the RI among those without influenza vaccine coadministration was 0.72 (95% CI: 0.61–0.86). For the 1–28 day risk interval, the RI among those with influenza vaccine coadministration was 0.95 (95% CI: 0.77–1.19) in the overall analyses, while the RI among those without influenza vaccine coadministration was 0.83 (95% CI: 0.73–0.94). Across age subgroups, we did not observe an increased risk of first-ever tinnitus overall or by influenza vaccine coadministration for both 1–14 and 1–28 day risk intervals.

### 3.3. First-in-1-year tinnitus

In the overall analyses of first-in-1-year tinnitus, the RI of first-in-1-year tinnitus following COVID-19 XBB.1.5 vaccination was 0.76 (95% CI: 0.67–0.87) in the 1–14 day risk interval and 0.90 (95% CI: 0.82–0.99) in the 1–28 day risk interval (Table 4). For the 1–14 day risk interval, the RI among those with influenza vaccine coadministration was 0.98 (95% CI: 0.76–1.26), while the RI among those without influenza vaccine coadministration was 0.70 (95% CI: 0.60–0.82). For the 1–28 day risk interval, the RI among those with influenza vaccine coadministration was 0.98 (95% CI: 0.80–1.19), while the RI among those without influenza vaccine coadministration was 0.88 (95% CI: 0.79–0.98). No increased risk was observed across age groups, and similar patterns were noted for individuals with and without influenza vaccine coadministration for both 1–14 and 1–28 day risk intervals.

In sensitivity analyses using extended risk intervals, we found no evidence of increased tinnitus risk following COVID-19 XBB.1.5 vaccination. In the overall analyses of first-ever tinnitus, the RI was 0.93 (95% CI: 0.85–1.02) for the 1–42 day risk interval and 0.91 (95% CI: 0.83–1.00) for the 1–90 day risk interval (Table S1). Similarly, in the overall analyses of first-in-1-year tinnitus, the RI was 0.95 (95% CI: 0.87–1.03) for the 1–42 day risk interval and 0.96 (95% CI: 0.89–1.04) for the 1–90 day risk interval (Table S2). Subgroup analyses by age and influenza vaccine coadministration on the same day also showed no elevated risk across both risk intervals.

Sensitivity analyses excluding tinnitus cases within 30 days of SARS-CoV-2 infection showed only slight differences in RIs (95% CI) in the overall analyses, across 1–14 and 1–28 day risk intervals, for first-ever and first-in-1-year tinnitus outcomes, with or without influenza vaccine coadministration (Table S3).

## 4. Discussion

Our study assessed the risk of tinnitus following administration of COVID-19 XBB.1.5 vaccines using a SCCS design. We examined tinnitus cases from inpatient, ED, and outpatient settings occurring between September 1, 2023, and March 31, 2024. Our analysis focused on individuals aged 12 years and older who received the Pfizer-BioNTech or Moderna COVID-19 XBB.1.5 vaccines, including those with and without influenza vaccine coadministration. Using the event-dependent SCCS approach, we compared incidences of first-ever and first-in-1-year tinnitus within predefined risk and control intervals while adjusting for seasonality. We excluded Day 0 when calculating RIs due to potential ambiguity in the temporal relationship between vaccination and tinnitus onset. Overall, our findings showed no association between COVID-19 XBB.1.5 vaccination and tinnitus risk, either in the general population or within age-specific subgroups.

Our findings are consistent with prior research examining tinnitus risk following COVID-19 vaccination. A retrospective chart review at a tertiary otolaryngology practice and found no evidence of increased tinnitus risk after COVID-19 vaccination [23]. Similarly, Yih et al. analyzed data from VAERS and the Vaccine Safety Datalink (VSD) and found comparable tinnitus incidence rates following COVID-19 and influenza vaccinations [22]. Despite these findings showing no association, there is still some debate in the scientific community. Some survey studies have found a potential association between COVID-19 vaccination and tinnitus. For instance, Wang et al. reported a higher frequency of tinnitus for COVID-19 vaccines (1.87 %) than for other vaccines (0.52 %) based on data in VAERS and a survey among those who experienced tinnitus after COVID-19 vaccination [24]. Tai et al. conducted a cross-sectional online survey with 1511 respondents who reported having COVID-19-related tinnitus [35]. The respondents were categorized into four groups: COVID-19 infection, long COVID, COVID-19 vaccination, and pre-existing tinnitus. It was found that 37.2 % participants reported tinnitus following COVID-19 vaccination while 44.9 % reported having tinnitus associated with pre-existing tinnitus, and tinnitus severity was found to be lower in the pre-existing tinnitus group than in any of the COVID-19-associated tinnitus groups. Additionally, Yellamsetty et al. assessed the impact of COVID-19 vaccination on the onset and severity of tinnitus in a survey of 372 individuals who reported new-onset tinnitus or worsening of pre-existing tinnitus following COVID-19 vaccination, finding no significant difference in tinnitus symptom onset between mRNA and non-mRNA COVID-19 vaccines [36]. In contrast to other survey-based studies, Saunders et al. did not find clear evidence supporting an association between COVID-19 vaccination and tinnitus [37]. Their large population-based survey, which compared symptom reports before and during the pandemic, suggested that inconsistent reporting patterns, recall bias, and psychosocial factors rather than vaccination may explain observed auditory symptoms. In addition, unlike rigorous epidemiological studies that carefully select comparator populations and appropriately adjust for confounders, these survey studies lacked a defined denominator and did not account for confounding, making them not well suited for population-based assessments of tinnitus risk after COVID-19 vaccination.

An Australian SCCS study reported a statistically significant association between mRNA COVID-19 vaccines and the presentation of tinnitus in a primary care setting [26]. In contrast, our study did not observe a similar association. Several methodological differences may explain this discrepancy. First, the Australian study included individuals aged 20–65 years and covered the period from January 1, 2021, to March 28, 2023, whereas our study included individuals aged ≥12 years and spanned from September 1, 2023, to March 31, 2024. Second, tinnitus cases in the Australian study were identified using SNOMED CT codes from general practice data, capturing first presentations in a primary care setting, while our study identified first-ever and first-in-1-year tinnitus cases across inpatient, emergency department, and outpatient settings, with most cases from outpatient visits. Third, although both studies excluded Day 0 events, our analysis used multiple risk intervals (1–14, 1–28, 1–42, and 1–90 days), while the Australian study used a single 1–42 day risk interval. Additionally, we used the event-dependent SCCS model and adjusted for seasonality by including month as a time-varying covariate, whereas the Australian study used standard SCCS, did not apply a washout window, and did not explicitly account for seasonal effects. Lastly, while the Australian study acknowledged SARS-CoV-2 infection as a potential confounder but did not adjust for it due to data limitations, we conducted sensitivity analyses excluding tinnitus events occurring within 30 days of a SARS-CoV-2 infection to mitigate this potential bias. These differences in study design, population, study period, and confounder adjustment likely contributed to the divergent findings.

Our study has several strengths. We used the event-dependent SCCS design that inherently controls time-invariant confounders including sex, race/ethnicity, and underlying comorbidities. We accounted for potential seasonality by including calendar month in the SCCS analyses. The inclusion of tinnitus cases from diverse clinical settings (inpatient, ED, and outpatient) improved the study’s comprehensiveness. Additionally, we conducted chart reviews to evaluate a random sample of cases occurring on Day 0, confirming that tinnitus onset preceded vaccination in these cases. Our study also benefited from accurate vaccination data and a large sample size within the KPSC system, supporting robust and reliable findings. Furthermore, we assessed tinnitus risk using both short (1–14 day and 1–28 day) and extended (1–42 day and 1–90 day) risk intervals, allowing for a more nuanced evaluation of potential temporal associations and enhancing the robustness of our conclusions.

During the study observation period, which spanned the 2023–2024 respiratory virus season, the predominant circulating COVID-19 variant in the United States was JN.1, a sublineage of the Omicron BA.2.86 variant. This variant became dominant in late 2023 and remained prevalent into early 2024. According to the CDC [38], JN.1 demonstrated increased transmissibility but did not appear to cause more severe disease compared to earlier variants. Importantly, the updated 2023–2024 COVID-19 vaccines offered protection against XBB and JN lineage hospitalizations [39].

Although SARS-CoV-2 infection status could be a potential confounder, we did not include it as a covariate in the primary analysis due to limitations in data completeness. The relatively mild clinical presentation of JN.1, combined with reduced testing rates and widespread use of at-home testing kits, likely led to underreporting of infections documented in EHR. To address this limitation, we conducted a sensitivity analysis excluding tinnitus events that occurred within 30 days of a documented SARS-CoV-2 infection in the overall analyses for first-ever and first-in-1-year tinnitus with 14- and 28-day risk intervals. The results of this sensitivity analysis were consistent with our primary findings, suggesting that the observed no-association findings were unlikely to be driven by concurrent or recent SARS-CoV-2 infection.

Despite its strengths, our study has some limitations. First, we relied on ICD-10 codes and electronic healthcare data to identify tinnitus cases without conducting chart reviews to confirm the diagnosis and determine the onset dates. Tinnitus event dates in the SCCS analyses were based on the diagnosis dates of medical encounter recorded in the EHR, rather than the actual date of symptom onset. As a result, there may be some temporal misclassification, particularly for individuals who delayed seeking care after symptom onset. This could lead to underestimation or attenuation of any true temporal association between vaccination and tinnitus onset. However, we expect this misclassification to be non-differential with respect to vaccination status and therefore unlikely to introduce systematic bias in the estimated risk. Second, while the overall sample sizes in the analyses were large, the sample sizes for subgroup analyses were modest. Third, the study was conducted within a single integrated healthcare system, which may limit the generalizability of findings to other insured or uninsured populations. Fourth, our study only captured medically attended tinnitus cases, potentially underestimating the true incidence if individuals with mild symptoms did not seek care [40]. Lastly, tinnitus severity and its impact on quality of life were not assessed, limiting our understanding of the condition’s clinical implications.

In conclusion, our study found no association between COVID-19 XBB.1.5 vaccination and tinnitus risk overall or within age-specific subgroups, regardless of coadministration with influenza vaccine. These findings provide reassuring evidence supporting the safety of COVID-19 vaccination with respect to tinnitus risk.

## Supplementary Material

SAS codes for preparing data for running dependent-SCCS in R

## Figures and Tables

**Table 1:** Demographic characteristics of recipients of COVID-19 XBB.1.5 vaccines who experienced a first-ever tinnitus event in inpatient, emergency department, and outpatient settings at Kaiser Permanente Southern California during the period from September 1, 2023 to March 31, 2024.

	Coadministered with influenza vaccine (*N* = 922)	Not coadministered with influenza vaccine (*N* = 13,018)	Overall (N = 13,940)
			
	n (%)	n (%)	n (%)

**Age (years)**			
12–39	164 (17.8)	2,362 (18.1)	2,526 (18.1)
40–59	274 (29.7)	4,207 (32.3)	4,481 (32.1)
60+	484 (52.5)	6,449 (49.5)	6,933 (49.7)
**Sex**			
Female	422 (45.8)	6,960 (53.5)	7,382 (53.0)
Male	500 (54.2)	6,056 (46.5)	6,556 (47.0)
Missing	0 (0.0)	2 (0.0)	2 (0.0)
**Race/ethnicity**			
Hispanic	325 (35.2)	5,559 (42.7)	5,884 (42.2)
Non-Hispanic White	372 (40.3)	4,599 (35.3)	4,971 (35.7)
Non-Hispanic Asian/Pacific Islander	109 (11.8)	1,394 (10.7)	1,503 (10.8)
Non-Hispanic Black	58 (6.3)	872 (6.7)	930 (6.7)
Other/Unknown	58 (6.3)	594 (4.6)	652 (4.7)
**Setting**			
Outpatient	909 (98.6)	12,612 (96.9)	13,521 (97.0)
Inpatient	2 (0.2)	73 (0.6)	75 (0.5)
Emergency department	11 (1.2)	333 (2.6)	344 (2.5)

**Table 2:** Demographic characteristics of recipients of COVID-19 XBB.1.5 vaccines who experienced a first-in-1-year tinnitus event in inpatient, emergency department, and outpatient settings at Kaiser Permanente Southern California during the period from September 1, 2023 to March 31, 2024.

	Coadministered with influenza vaccine (*N* = 1,144)	Not coadministered with influenza vaccine (*N* = 16,731)	Overall (*N* = 17,875)
			
	n (%)	n (%)	n (%)

**Age (years)**			
12–39	179 (15.6)	2,655 (15.9)	2,834 (15.9)
40–59	337 (29.5)	5,200 (31.1)	5,537 (31.0)
60+	628 (54.9)	8,876 (53.1)	9,504 (53.2)
**Sex**			
Female	529 (46.2)	8,944 (53.5)	9,473 (53.0)
Male	615 (53.8)	7,785 (46.5)	8,400 (47.0)
Missing	0 (0.0)	2 (0.0)	2 (0.0 %)
**Race/ethnicity**			
Hispanic	405 (35.4)	7,166 (42.8)	7,571 (42.4)
Non-Hispanic White	458 (40.0)	5,914 (35.3)	6,372 (35.6)
Non-Hispanic Asian/Pacific Islander	140 (12.2)	1,794 (10.7)	1,934 (10.8)
Non-Hispanic Black	74 (6.5)	1,164 (7.0)	1,238 (6.9)
Other/Unknown	67 (5.9)	693 (4.1)	760 (4.3)
**Setting**			
Outpatient	1127 (98.5)	16,223 (97.0)	17,350 (97.1)
Inpatient	3 (0.3)	94 (0.6)	97 (0.5)
Emergency department	14 (1.2)	414 (2.5)	428 (2.4)

**Table 3:** Relative incidences (95% confidence intervals) of first-ever tinnitus event in inpatient, emergency department, and outpatient settings after COVID-19 XBB.1.5 vaccination during the period from September 1, 2023 to March 31, 2024.

	1–14 day risk interval				1–28 day risk interval			
		
	Overall	12–39 years	40–59 years	60+ years	Overall	12–39 years	40–59 years	60+ years

Overall	0.78 (0.67–0.90)	0.94 (0.60–1.46)	0.77 (0.57–1.05)	0.76 (0.63–0.91)	0.87 (0.78–0.96)	0.93 (0.66–1.31)	0.88 (0.70–1.10)	0.86 (0.75–0.98)
Coadministered with influenza vaccine	0.93 (0.70–1.24)	0.76 (0.35–1.66)	1.01 (0.61–1.67)	0.96 (0.65–1.42)	0.95 (0.77–1.19)	0.68 (0.37–1.23)	0.98 (0.66–1.45)	1.05 (0.79–1.40)
Not coadministered with influenza vaccine	0.72 (0.61–0.86)	1.02 (0.59–1.76)	0.66 (0.45–0.98)	0.71 (0.58–0.87)	0.83 (0.73–0.94)	1.07 (0.70–1.64)	0.82 (0.63–1.08)	0.81 (0.70–0.94)

**Table 4:** Relative incidences (95% confidence intervals) of first-in-1-year tinnitus event in inpatient, emergency department, and outpatient settings after COVID-19 XBB.1.5 vaccination during the period from September 1, 2023 to March 31, 2024.

	1–14 day risk interval				1–28 day risk interval			
		
	Overall	12–39 years	40–59 years	60+ years	Overall	12–39 years	40–59 years	60+ years

Overall	0.76 (0.67–0.87)	0.93 (0.60–1.43)	0.77 (0.58–1.02)	0.74 (0.64–0.87)	0.90 (0.82–0.99)	0.89 (0.64–1.25)	0.89 (0.73–1.09)	0.91 (0.82–1.02)
Coadministered with influenza vaccine	0.98 (0.76–1.26)	0.73 (0.33–1.58)	1.06 (0.68–1.66)	1.02 (0.73–1.43)	0.98 (0.80–1.19)	0.64 (0.35–1.17)	1.05 (0.74–1.49)	1.05 (0.81–1.35)
Not coadministered with influenza vaccine	0.70 (0.60–0.82)	1.02 (0.61–1.73)	0.65 (0.46–0.93)	0.69 (0.58–0.82)	0.88 (0.79–0.98)	1.03 (0.68–1.56)	0.82 (0.64–1.05)	0.89 (0.78–1.00)

## Data Availability

Data will be made available on request.

## Appendix A. Supplementary data

Supplementary data to this article can be found online at https://doi.org/10.1016/j.vaccine.2025.127548.
