# Supplementary material for: Tinnitus risk after COVID-19 XBB.1.5 vaccination: A self-controlled case series study
Source: Vaccine. Author manuscript; Available in PMC 2026 Jul 10. (PMC13354291; doi:10.1016/j.vaccine.2025.127548)
Supplement: SAS codes for preparing data for running dependent-SCCS in R [file NIHMS2184492-supplement-SAS_codes_for_preparing_data_for_running_dependent-SCCS_in_R.docx]

**APPENDICES**

**SAS codes for preparing data for running dependent-SCCS in R**

/****************************************************************************

* Title: SAS codes for preparing the data for running dependent-SCCS in R

* Developer: Vennis Hong and Stanley Xu

* Research and Evaluation

* Kaiser Permanente Southern California

* Email: stan.xu@kp.org

* Date Created: 07/04/2025

****************************************************************************/

libname in 'S:\R&E Scientists\Stanley Xu\tinnitus';

libname out 'S:\R&E Scientists\Stanley Xu\ tinnitus \for_R';

/***********************************************************************************************************

*The case only dataset, in.tin_first_ever_ira and in.tin_first_1yr_ira, have first-ever and first-in-1-*year tinnitus events in IP, ED and OP settings during the study period, 9/1/2023-3/31/2024, *among those aged >=12 *years. The datasets have the following variables:

*case, a unique number for each case

*age, age on 9/1/2023, gender, race/ethnicity, sex

* stop, date of disenrollment

* group for vaccination status, a flag for coadministration and was used to make the table 1

*xbb1, the date when the 1st dose covid-19 xbb vaccine was given

*xbb2, the date when the 2nd dose covid-19 xbb vaccine was given

* concom_flu, an indicator whether influenza vaccine was given on the same day

* fup_start, a SAS date of 9/1/2023

* fup_end, a SAS date of 3/31/2024 or disenrollment date, whichever came earlier

*admitdate, date when a tinnitus event occurred

*death_date, date of death

*******************************************************************************************************/

************ Output overall analytical datasets **************;

%let index_date.=mdy (9,1,2023);

%let end_date.=mdy (3,31,2024);

%macro analytic_datasets(in);

data out.&in._overall;

set &in.;

age_grp = put(age, age.);

case+1;

fup_start = &index_date.;

fup_end = min(&end_date., xbb2-1, deathdate, stop);

vac_days = xbb1-fup_start+1;

event_days = admitdate-fup_start+1;

event_vac_days = admitdate-xbb1+1;

fup_start_days = 1;

fup_end_days = fup_end-fup_start+1;

format fup_start fup_end mmddyy10.;

run;

%mend;

%analytic_datasets(tin_first_ever_ira);

%analytic_datasets(tin_first_1yr_ira);

*************** Output overall by age *************;

%macro overall_age(in);

data out.&in._Under40 out.&in._40_59 out.&in._Over60;

set out.&in._overall;

if age_grp='Under 40' then output out.&in._Under40;

else if age_grp='40-59' then output out.&in._40_59;

else if age_grp='60+' then output out.&in._Over60;

run;

%mend;

%overall_age(tin_first_ever_ira);

%overall_age(tin_first_1yr_ira);

*************** Output overall by concomitant flu *************;

%macro overall_flu(in);

data out.&in._Flu out.&in._NoFlu;

set out.&in._overall;

if concom_flu=1 then output out.&in._Flu;

else if concom_flu=0 then output out.&in._NoFlu;

if missing(xbb1) then output out.&in._Flu;

run;

%mend;

%overall_flu(tin_first_ever_ira);

%overall_flu(tin_first_1yr_ira);

*************** Output overall by age and concomitant flu *************;

%macro overall_flu(in);

data out.&in._Flu_Under40 out.&in._Flu_40_59 out.&in._Flu_Over60;

set out.&in._Flu;

if age_grp='Under 40' then output out.&in._Flu_Under40;

else if age_grp='40-59' then output out.&in._Flu_40_59;

else if age_grp='60+' then output out.&in._Flu_Over60;

run;

data out.&in._NoFlu_Under40 out.&in._NoFlu_40_59 out.&in._NoFlu_Over60;

set out.&in._NoFlu;

if age_grp='Under 40' then output out.&in._NoFlu_Under40;

else if age_grp='40-59' then output out.&in._NoFlu_40_59;

else if age_grp='60+' then output out.&in._NoFlu_Over60;

run;

%mend;

%overall_flu(tin_first_ever_ira);

%overall_flu(tin_first_1yr_ira);

**R codes for fitting event-dependent SCCS with 1-14 day risk interval for first-ever tinnitus**

#for read sas dataset

install.packages("haven")

library(dplyr)

library(SCCS)

library(haven)

library(knitr)

# Overall

overall <- read_sas("tin_first_ever_ira_overall.sas7bdat")

under40 <- read_sas("tin_first_ever_ira_under40.sas7bdat")

age40_59 <- read_sas("tin_first_ever_ira_40_59.sas7bdat")

over60 <- read_sas("tin_first_ever_ira_over60.sas7bdat")

# Had concomitant flu

flu <- read_sas("tin_first_ever_ira_flu.sas7bdat")

flu_under40 <- read_sas("tin_first_ever_ira_flu_under40.sas7bdat")

flu_age40_59 <- read_sas("tin_first_ever_ira_flu_40_59.sas7bdat")

flu_over60 <- read_sas("tin_first_ever_ira_flu_over60.sas7bdat")

# No concomitant flu

noflu <- read_sas("tin_first_ever_ira_noflu.sas7bdat")

noflu_under40 <- read_sas("tin_first_ever_ira_noflu_under40.sas7bdat")

noflu_age40_59 <- read_sas("tin_first_ever_ira_noflu_40_59.sas7bdat")

noflu_over60 <- read_sas("tin_first_ever_ira_noflu_over60.sas7bdat")

seas <- cumsum(c(30, 31, 30, 31, 31, 29, 31))

sccs_output <- function(indata, rw, analyses) {

eventdepenexp(indiv=case, astart=fup_start_days, aend=fup_end_days,

aevent=event_days, adrug=vac_days,

aedrug=vac_days+rw, expogrp=c(0,1),

sameexpopar=F, agegrp=seas,

data=indata)[2] %>%

as.data.frame(row.names=NULL) %>%

select(conf.int.exp.coef., conf.int.lower..95, conf.int.upper..95) %>%

filter(row_number()==2) %>%

mutate(RW = rw, .before = conf.int.exp.coef.) %>%

mutate(analyses = analyses, .before = RW)

}

## 14-day risk window, Overall analyses

p1 <- sccs_output(overall, 14, 'Overall')

p2 <- sccs_output(flu, 14, 'Overall flu')

p3 <- sccs_output(noflu, 14, 'Overall no flu')

p4 <- rbind(p1, p2, p3)

kable(p4)

# 14-day risk window, Under 40 analyses

p5 <- sccs_output(under40, 14, 'Under 40')

p6 <- sccs_output(flu_under40, 14, 'Under 40 flu')

p7 <- sccs_output(noflu_under40, 14, 'Under 40 no flu')

p8 <- rbind(p5, p6, p7)

kable(p8)

#14-day risk window, 40-59 analyses

p9 <- sccs_output(age40_59, 14, '40-59')

p10 <- sccs_output(flu_age40_59, 14, '40-59 flu')

p11 <- sccs_output(noflu_age40_59, 14, '40-59 no flu')

p12 <- rbind(p9, p10, p11)

kable(p12)

#14-day risk window, Over 60 analyses

p13 <- sccs_output(over60, 14, 'Over 60')

p14 <- sccs_output(flu_over60, 14, 'Over 60 flu')

p15 <- sccs_output(noflu_over60, 14, 'Over 60 no flu')

p16 <- rbind(p13,p14,p15)

kable(p16)

**Table S1**. Relative incidences (95% confidence intervals) of first-ever tinnitus event in inpatient, emergency department, and outpatient settings 42 and 90 days after COVID-19 XBB.1.5 vaccination during the period from September 1, 2023 to March 31, 2024.

|  | 1–42 day risk interval | | | | 1–90 day risk interval | | | |
| --- | --- | --- | --- | --- | --- | --- | --- | --- |
|  | Overall | 12-39 years | 40-59 years | 60+ years | Overall | 12-39 years | 40-59 years | 60+ years |
| Overall | 0.93 (0.85-1.02) | 0.95 (0.78-1.16) | 0.88 (0.70-1.10) | 0.93 (0.83-1.04) | 0.91 (0.83-1.00) | 0.98 (0.71-1.34) | 0.94 (0.78-1.14) | 0.90 (0.81-1.00) |
| Coadministered with influenza vaccine | 0.95 (0.79-1.16) | 1.02 (0.72-1.45) | 0.98 (0.66-1.45) | 1.03 (0.80-1.34) | 0.90 (0.75-1.09) | 0.65 (0.39-1.07) | 1.08 (0.76-1.54) | 0.90 (0.71-1.15) |
| Not coadministered with influenza vaccine | 0.91 (0.82-1.02) | 0.90 (0.70-1.14) | 0.82 (0.63-1.08) | 0.91 (0.80-1.03) | 0.91 (0.82-1.01) | 1.24 (0.82-1.90) | 0.88 (0.70-1.11) | 0.91 (0.80-1.02) |
|  |  |  |  |  |  |  |  |  |

**Table S2**. Relative incidences (95% confidence intervals) of first-in-1-year tinnitus event in inpatient, emergency department, and outpatient settings 42 and 90 days after COVID-19 XBB.1.5 vaccination during the period from September 1, 2023 to March 31, 2024.

|  | 1–42 day risk window | | | | 1–90 day risk window | | | |
| --- | --- | --- | --- | --- | --- | --- | --- | --- |
|  | Overall | 12-39 years | 40-59 years | 60+ years | Overall | 12-39 years | 40-59 years | 60+ years |
| Overall | 0.95 (0.87-1.03) | 0.92 (0.67-1.24) | 0.94 (0.79-1.13) | 0.96 (0.87-1.06) | 0.96 (0.89-1.04) | 0.96 (0.70-1.30) | 0.99 (0.83-1.18) | 0.97 (0.89-1.06) |
| Coadministered with influenza vaccine | 0.99 (0.84-1.18) | 0.65 (0.39-1.09) | 1.03 (0.75-1.42) | 1.08 (0.86-1.36) | 0.97 (0.82-1.14) | 0.65 (0.40-1.06) | 1.17 (0.85-1.61) | 0.98 (0.79-1.21) |
| Not coadministered with influenza vaccine | 0.93 (0.85-1.02) | 1.07 (0.73-1.58) | 0.90 (0.72-1.12) | 0.94 (0.84-1.04) | 0.96 (0.88-1.05) | 1.19 (0.80-1.79) | 0.92 (0.74-1.13) | 0.97 (0.88-1.07) |

**Table S3**. Relative incidences (95% confidence intervals) of tinnitus in inpatient, emergency department, and outpatient settings 14 and 28 days after COVID-19 XBB.1.5 vaccination during the period from September 1, 2023 to March 31, 2024 after excluding those occurring within 30 days of SARS-CoV-2 infection.

| Outcome |  | 1–14 day risk interval | 1–28 day risk interval |
| --- | --- | --- | --- |
| First-ever tinnitus | Overall | 0.78 (0.67-0.91) | 0.88 (0.79-0.98) |
|  | Coadministered with influenza vaccine | 0.92 (0.68-1.23) | 0.95 (0.76-1.19) |
|  | Not coadministered with influenza vaccine | 0.73 (0.62-0.87) | 0.85 (0.75-0.96) |
| First-in-1-year tinnitus | Overall | 0.77 (0.67-0.88) | 0.92 (0.84-1.01) |
|  | Coadministered with influenza vaccine | 0.97 (0.75-1.26) | 0.98 (0.80-1.20) |
|  | Not coadministered with influenza vaccine | 0.71 (0.61-0.83) | 0.89 (0.80-1.00) |
